# Financial burden of healthcare for cancer patients with social medical insurance: a multi-centered study in urban China

**DOI:** 10.1186/s12939-017-0675-y

**Published:** 2017-10-10

**Authors:** Wenhui Mao, Shenglan Tang, Ying Zhu, Zening Xie, Wen Chen

**Affiliations:** 1 School of Public Health, Fudan University, P.O. Box 187, 138 Yi Xue Yuan Road, Shanghai, 200032 China; 2 Duke Global Health Institute, Duke University, 310 Trent Drive, Durham, NC 27710 USA; 3 Duke Kunshan University, No. 8 Duke Avenue, Kunshan, Jiangsu Province 215316 China

**Keywords:** Cancer care, Health insurance, Out-of-pocket (OOP), Financial protection

## Abstract

**Background:**

Cancer accounts for one-fifth of the total deaths in China and brings heavy financial burden to patients and their families. Chinese government has made strong commitment to develop three types of social medical insurance since 1997 and recently, more attempts were invested to provide better financial protection.

To analyze health services utilization and financial burden of insured cancer patients, and identify the gaps of financial protection provided by insurance in urban China.

**Methods:**

A random sampling, from Urban Employee's Basic Medical Insurance claim database, was performed in 4 cities in 2008 to obtain insurance claim records of cancer patients. Services utilization, medical expenses and out-of-pocket (OOP) payment were the metrics collected from the insurance claim database, and household non-subsistence expenditure were estimated from Health Statistics. Catastrophic health expenditure was defined as household’s total out-of-pocket payments exceed 40% of non-subsistence expenditure. Stratified analysis by age groups was performed on service use, expenditure and OOP payment.

**Results:**

Data on 2091 insured cancer patients were collected. Reimbursement rates were over 80% for Shanghai and Beijing while Fuzhou and Chongqing only covered 60%–70% of total medical expenditure. Shanghai had the highest reimbursement rate (88.2%), high total expenditure ($1228) but lowest OOP payment ($170) among the four cities. Chongqing and Fuzhou's insured cancer patients exclusively preferred tertiary hospitals for outpatient services. Fuzhou led the annual total medical expense ($9963), followed by Chongqing, Beijing and Shanghai. The average OOP as proportion of household’s capacity to pay was 87.3% (Chongqing), 66.0% (Fuzhou), 33.7% (Beijing) and 19.6% (Shanghai). Elderly insured cancer patients utilized fewer outpatient services, had lower number of inpatient admissions but longer length of stay, and higher total expenditure.

**Conclusions:**

Social economic development was not necessarily associated with total medical expense but determined the level of financial protection. The economic burden of insured cancer patients was reduced by insurance but it is still necessary to provide further financial protections and improve affordability of healthcare for cancer patients in China.

## Background

Cancer, accounting for 22.32% of the total deaths in China, is the second most common cause of death [1]. In 2012, there were 2.8 million incident cases and 1.96 million deaths from cancer in China, constituting to approximately a quarter of all cancer deaths in the world [1]. Yet the health investment per cancer patient was merely $2202 in China, while it totals to $37,837 and $86,759 for UK and US, respectively. With advancement of technologies, an increase in the medical expense of cancer treatment is expected. In 2012, the average cost for a Chinese patient with non-small cell lung cancer was $16,955 in last 3 months of life [2], equivalent to 57% of the annual household disposable income for an affluent urban household from Eastern China, or 82% of a poor urban family from Central China [2].

Without a sustainable financing mechanism, the burden of cancer treatment would escalate devastatingly for patients and families, and eventually lead to severe social inequity. To improve the access to healthcare, the Chinese government made noticeable commitment to establish the Social Health Insurance since last two decades and many efforts were invested to further improve the benefit package of financial protection provided by insurance.

In 1997, Urban Employees’ Basic Medical Insurance Scheme (UEBMI) was first established, compulsorily covering employees (and retirees) in urban areas through shared premium between employees and employers. With the relatively sufficient financing sources, UEBMI developed personal medical account and pooling of funds to cover both inpatient and outpatient services. Later in 2003, New Cooperative Medical Scheme (NCMS) scaled up quickly in rural regions and provided very basic benefit package to rural residents by collecting premium from rural families and the government subsidies. NCMS only focused on the reimbursement of inpatient services due to limited financing sources at the very beginning [3]. Urban Residents’ Basic Medical Insurance Scheme (URBMI) was piloted in 2007 and aimed to cover residents in urban areas. With similar institutional settings to NCMS, URBMI collected premium from enrollees, received government subsidies, and provided relatively lower reimbursement on inpatient and outpatient services than UEBMI. Although NCMS and URBMI are voluntarily insurance, the local Human Resources and Social Security Department or Health and Family Planning Department managed NCMS and URBMI and National, Provincial and local government provide lots of subsidies to support their operation. Besides UEBMI, URBMI and NCMS, the Civil Affair Department also established Medical Financial Assistance and perform as safe-net to people live in poverty.

UEBMI, URBMI and NCMS, as the Social Health Insurance (SHI) system of China, covered over 95% of the Chinese population by the end of 2012 [3]. Within the overall policy frame set by the central government, the operational supervision of UEBMI, URBMI and NCMS is at the discretion of prefecture- or county-level city.

With the reimbursement from the three insurance schemes, China made the greatest reduction in Out-of-Pocket payments (OOPs) from 2000 to 2012 among all of the countries in Asia-Pacific region [4]. However, the share of OOPs still took up over 30% in Total Health Expenditure (THE) [3] due to limited services covered and relatively low co-payment provided by SHIs, especially in the rural regions of China. According to the National Household Health Survey, the reimbursement rate for hospital admission was 63.2% and 26.6% for UEBMI and NCMS patients, respectively, in 2008. In 2012, the gap was narrowed but still existed that the reimbursement rate was 68.8% for UEBMI patients and 50.1% for NCMS patients [5, 6].

Many endeavors have been invested to improve the financial protection of insurance. First, after having a stable number of enrollees, NCMS and URBMI gradually increased the reimbursement rate on inpatient services. Secondly, more and more places provide reimbursement for outpatient services even with relatively low reimbursement rate. Thirdly, in most cities, severe diseases, such as cancer or urinaemia, are entitled to additional reimbursement for outpatient services [7]. Fourthly, URBMI and NCMS have been merged in an increasingly number of provinces in China which also have increased the risk sharing capacity. Fifthly, the State Council issued the “Opinions on the Full Implementation of the Insurance Program of Catastrophic Diseases (IPCD) for Urban and Rural Residents” in 2015 and IPCD was expected to improve the financial protection from insurance [8].

However, few published research reported whether these initiatives actually reduced the financial burden of patients. In addition, the direct economic burden of chronic diseases with high medical expense such as cancer in a period of time was under-reported [9]. We herein focus on Urban Employees’ Basic Medical Insurance (UEBMI) which had a national database in standardized format and a better benefit package than that of the other two social health insurance schemes (URBMI and NCMS). Our research question is whether UEBMI provided sufficient financial protection on cancer patients and what experiences can be learnt for URBMI and NCMS in developing benefit package.

## Methods

### Selection criteria for study sites

Study sites were selected based on the following criteria: 1) locate in different part of China 2) have innovative reimbursement policy for cancer patients 3) are the provincial capital or municipal’s with adequate medical resources and can be regarded as the regional medical center 4) have at least one cancer specialized hospitals. Four cities, Shanghai, Beijing, Fuzhou and Chongqing, were selected.

Shanghai, located in the east coast of China, has the highest GDP per capita in China ($10,530^1^ in 2008). Beijing is the capital city of China located in Northern China and also has high level of economic development. Fuzhou, as the provincial capital of Fujian Provinces, is located in Southern China with above average GDP per capita and Chongqing is one of the most important medical centers in the Western part of China.

The UEBMI policies also had different features in the four cities. Shanghai and Beijing had technical and personnel supremacy in medical practices and both Shanghai and Beijing citizens were bestowed a higher contribution in financing of UEBMI, which brought a better benefit package. More specifically, more services were covered and the reimbursement rate and the maximum payment limit were higher. For OP visits of insured cancer patients. Shanghai had a broad services list and no deductible payment, while Beijing regarded OP visits in 3 months as one hospital admission and applied identical coverage scheme as for hospital admission. Fuzhou required a $144 deductible payment, and set up different reimbursement rates for different levels of hospital, and Chongqing required OP treatment for cancer the same deductible as hospital admission but employed a fixed reimbursement rate. The provider payment methods have also been developed in different ways. Shanghai adopted the Global Budget, while services charge remained as the main payment method in Beijing. Fuzhou and Chongqing set a fixed quota of reimbursement for each hospital admission. However, when the actual expense was lower than the quota, hospitals in Fuzhou were paid the actual expenses, but the hospitals in Chongqing were paid the quota (Table 1).

**Table 1:** Insurance Policy in Study Cities(2008)

Shanghai [21]	Beijing [22]	Fuzhou [23]	Chongqing [24]
Financing contribution: Format: basic insurance + supplementary insurance (as proportion of annual income)			
12% + 2%	11% + $5 &1%	10%	10% + $4 &1%
Personal Medical Account			
Op visit, deductible and copayment for hospital admission and retail pharmacies			
OP visit coverage (after personal medical account): Format: deductible + reimbursement rate (for primary/secondary/tertiary hospital)			
Employee: $216 + 70%/60%/50% Retiree: Retired before 2001: $43 + 90%/85%/80% Retired after 2001:$101 + 55%–85%/50%–80%/45%–75% (vary to age)	Employee: $259 + 70%/50%/50% Retiree: $187CNY + 70–80%(vary to age) [25]	No coverage	No coverage
Hospital admission coverage: Format: deductible + reimbursement rate (for primary/secondary/tertiary hospital)			
Employee: $216 + 85% Retiree benefit: Retirement before 2001: $101 + 92% Retired after 2001: $173 + 92%	Deductible: 10% of average wage of urban employees from previous year; 5% from the second admission on; Reimbursement: From deductible to $4323, 90%/ 87%/85%; From $4323–$5764, 95%, 92%, and 90%; From $5764 to maximal payment limit, 97%/97%/95%	Employee: $86/$86/$115 + 85–92% Retiree: $86/$86/$115 + 90%–95% ^a^Deductible reduce $29 per hospital admission for multiple admissions	Deductible: 5%/8%/11% of average wage of urban employees from previous year; Reimbursement: From deductible to $720, 70–85%(vary to age); From $720–$1441, 75–90% (vary to age); From $1441–$4611, 80–95% ^b^Deductible decrease 1% per hospital admission for multiple admissions
Special management for malignant cancers (patients side)			
85% reimbursement for OP treatment of chemotherapy, radiotherapy, isotope therapy, interventional anti-cancer treatment, TCM anti-cancer treatment and necessary relevant examinations [26]	For OP treatment of radiotherapy and chemotherapy for malignant cancer, all related OP visits in 3 months were regarded as one hospital admission and has the same reimbursement policy	For chemotherapy and radiotherapy for malignant cancers, $144 deductible +90%/85%/85% (employee) or 94%/90%/90% (retiree) [27]	Same deductible as hospital admission + 90% reimbursement for radiotherapy, chemotherapy, and pain management [28]
Maximal payment limit			
$10,086 (including OP visit and hospital admission)	400% average wage of urban employees from previous year (including hospital admission and special OP visit)	$6484 (including hospital admission and special OP visit)	$4661 (including hospital admission and special OP visit)
Supplementary insurance			
Reimbursement rate 80% after maximal payment limit	Reimbursement rate 70% after maximal payment limit, with the annual maximal of $14,409 ($2882 for OP visit)	None	Reimbursement rate 100% after maximal payment limit, with the annual maximal of $57,637 [29]
Provider payment system^a^			
Global budget: every year, the insurance agency will negotiate with each hospital about its certain budget on the basis of its total expenditure in the past 3 years and the expanding trend of its services. If the actual expenditure was less than 80% of the budget, hospital will get the actual expenditure from the insurance fund; if the actual expenditure was around 80–100% of the budget, hospital will get the budget and the gap between the budget and the actual expense can be invested on the hospital (any forms of bonus for hospital employees are forbidden); if the actual expenditure was around 100–120% of the budget, the hospital can get the budget and part of the expense over the budget (the proportion depends on the operation of insurance of that year)	Service charge (case payment was explored in Beijing from 2009)	Certain package for each episode of hospital admission (also apply to OP visit of cancer patients): $893 per hospital admission for general disease. $2882 per hospital admission for 16 kinds of severe diseases (cancer included). For expense less than $2882, the hospital will get the actual expense	Certain package for each episode of hospital admission (also apply to OP visit of cancer patients): $836/$634 ~ $648/$432/$288 per hospital admission for teaching/tertiary/secondary/primary hospital; if the actual expense was around 80–100% of the contracted quota, the hospital will get the contracted quota

^a^Specific policy documents were not available, and the information was summarized from qualitative interviews with officers in insurance agencies

### Data collection

We collected both primary and secondary data for this research. Characters of patients and their medical expenditures were collected from primary data, and the social economic indicators were collected from secondary data.

UEBMI enrollees were required to register with the local Human Resources and Social Security Department to be eligible to the additional reimbursement policy for specific diseases such as cancer and urinaemia. A simple random sampling with 600 individuals in each city was performed on the registered UEBMI enrollees with cancer. For the consideration of the privacy of our study population, only the ID number, gender, primary diagnosis and age of the sample population was extracted. The ID number was then used to link to the UEBMI claim database. All records of study population’s hospital admissions and outpatient visits in 2008 in the UEBMI claim database were extracted. Indicators such as date of hospital admission and discharge (for hospital admission), date of outpatient visit, level of hospital, total medical insurance expense, and reimbursement from insurance were collected.

We used secondary data as a supplementary source to estimate the household income due to lack of information at individual level. More specifically, average consumption expenditure per capita, average size of household, average food expenditure per capita and/or Engel’s coefficient, average salary for employees and average pension for retirees in 2008 were collected from each city’s statistic yearbook.

### Key variables and analysis

Gender, average age, and types of cancer were analyzed to describe the demographic characters of the sample patients in different cities.

Average number of visits per year, proportion of different levels of hospitals, average length of stay were calculated to map the services utilization pattern of insured cancer patients in different cities.

Direct medical expenditure was analyzed from two perspectives. To assess the burden of each hospital admission and outpatient visit, average total expense per visit, average OOP payments and average reimbursement rate were analyzed. We also aggregated all the service utilizations and expenses of each patient in 2008 and got the burden of cancer treatment in one year, including average annual expense per patient, average annual OOP payments and average annual reimbursement rate per patient per year. Expenses of hospital admission and outpatient visit were analyzed separately first, and then summed to get the total annual expenditure. In additionally, average percentage of hospital admission expenditure to total medical expenditure was used to describe the proportion of medical expenditure from hospital admission.

Stratified analysis on two age groups, <60 and ≥60, was performed on service utilization and expenditure for further comparison [10].

Proportion of households experiencing catastrophic health expenditure was one of the key indicators to assess the financial burden of patients. According to WHO’s definition, the health expenditure becomes catastrophic when a household’s total out-of-pocket health payments exceed 40% of non-subsistence expenditure or household capacity to pay (CTP) [11]. To acquire this indicator, we used average consumption expenditure per capita, average size of household, average food expenditure per capita and/or Engel’s coefficient from yearbook to get household non-subsistence expenditure:

Household non-subsistence expenditure = average size of household *(average consumption expenditure per capita –average food expenditure per capita).

or = average size of household *average consumption expenditure per capita *(1-Engel’s coefficient).

Proportion of OOP as to household non-subsistence expenditure = total OOP payments/household non-subsistence expenditure.

## Results

### Characteristics of study population

The gender composition was generally balanced among study cities, with an average age of 63. Shanghai and Beijing had more middle aged patients while Chongqing had a higher proportion of patients over 60 years old (Table 2). The most prevalent malignant cancers across the four cities were similar, and were consistent with the national statistics. Bronchioles and lung cancer were the most prevalent in 4 cities [12], followed by breast cancer and digestive cancers. With the exception of breast cancer, the elderly population^2^ made up over 50% of cancer cases for each cancer type (Table 3).

**Table 2:** Demographic characteristics of cancer patients

	Shanghai	Beijing	Fuzhou	Chongqing
Sample size	600	600	600	608
# UEBMI patients (eligible)^a^	572	561	350	608
Gender Composition (%)				
Male	48.1	54.2	51.8	56.9
Average age (year, mean ± SD)	63.5 ± 12.4	63.2 ± 13.5	67.1 ± 10.0	67.4 ± 13.7
Age group (%)				
<40	1.7	5.5	0.3	2.3
40 ~ 59	40.2	34.2	23.7	26.5
60 ~ 79	47.6	50.8	66.8	53.6
>80	10.5	9.4	9.3	17.6

^a^We initially collected around 600 individuals in each city’s insurance database. However, the UEBMI and URBMI database was operated together in Shanghai, Beijing and Fuzhou. URBMI enrollers were removed and only UEBMI beneficiaries were included for final analysis

**Table 3:** The top 5 malignant cancers and its proportion in each study city (%)

City	Type of the Cancer	Proportion in the study city (%)	Proportion of elderly for this cancer (%)
Shanghai	Bronchioles and lung	14.2	64.2
	Breast	12.1	29.0
	Stomach	9.6	50.9
	Colon	9.1	78.8
	Rectum	4.0	65.2
	Subtotal	49.0	55.7
Beijing	Bronchioles and lung	22.1	71.8
	Breast	10.2	29.8
	Liver and intrahepatic bile duct	7.0	59.0
	Colon	6.8	81.6
	Stomach	4.8	59.3
	Subtotal	50.9	61.8
Fuzhou	Bronchioles and lung	20.0	80.0
	Stomach	16.3	80.7
	Liver and intrahepatic bile duct	12.0	83.3
	Breast	9.1	46.9
	Colon	8.3	82.8
	Subtotal	65.7	76.5
Chongqing	Bronchioles and lung	24.8	80.8
	Breast	8.1	53.1
	Liver and intrahepatic bile duct	6.7	64.1
	Colon	6.6	82.5
	Rectum	6.1	78.4
	Subtotal	52.3	74.4

### Use of inpatient services

The average number of hospital admissions per patient was 3 in Shanghai, Fuzhou and Chongqing, whereas it was significantly lower (1.9) in Beijing. The average length of stay ranged from 13 days in Shanghai to 24 days in others but the annual length of stay was similar in Shanghai and Beijing. Over 95% of hospital admission in Fuzhou and Chongqing were in tertiary hospitals whereas tertiary hospitals provided 60 ~ 80% of inpatient services in Shanghai and Beijing. The average total expense per admission shared a similar trend with the length of stay. The average expense per day, reflecting the intensity of services, on the other hand, showed a close correlation to the local socioeconomic status. Shanghai had the shortest length of stay, the least total expense and OOP payments. Beijing had similar length of stay as Chongqing but had the highest average total expense per admission. The reimbursement rate for hospital admission was higher in Shanghai and Beijing than that in Chongqing and Fuzhou (Table 4).

**Table 4:** Utilization and expense of hospital admission and OP visit in 2008

Service Type	Use of inpatient services				Use of outpatient services			
City	Shanghai	Beijing	Fuzhou	Chongqing	Shanghai	Beijing	Fuzhou	Chongqing
# patients	572	561	355	608	503	379	228	341
# of visits	1762	1058	1151	1747	11,523	10,132	4650	5096
Average number of visits per year	3.1	1.9	3.2	3.0	22.9	26.7	20.4	14.9
Per Hospital Admission/ Per OP visit								
Level of hospital (%)								
Tertiary	63.2	81.6	96.4	95.3	65.8	66.5	78.6	97.4
Secondary/Primary	36.8	18.4	3.6	4.7	34.2	33.5	21.4	2.6
Average length of stay (days)	13.7	24.0	21.3	24.9	/	/	/	/
Average total expense($)^a^	1608	3703	2888	2555	56	/^b^	46	80
Average OOP payments($)	255	518	695	895	7	/	14	24
Average reimbursement rate (%)	83.3	88.7	74.5	63.8	89.9	/	67.1	64.7
Average expense per day($)	117	154	136	103	/	/	/	/
Per Patient								
Average annual days of hospital admission	42.1	45.3	68.6	71.3	/	/	/	/
Average annual expense($)	4953	6984	9365	7340	1288	1336	930	1200
Average annual OOP payments($)	785	976	2252	2572	170	593	276	355
Average annual reimbursement rate (%)	83.8	87.7	74.7	61.5	88.2	50.1	50.1	47.8

^**a**^All currency was presented as the price of 2008 in US dollar (1USD = 6.94 CNY), no further discount applied

^**b**^All of the OP visit expense for cancer in 3 months were recorded as one hospital admission (Table 1), it is not applicable to get information of each OP visit in Beijing

### Use of outpatient services

Insured patients in Beijing led the average number of OP visit, which was 26.7 times per year, followed by Shanghai (22.9), Fuzhou (20.4) and Chongqing (14.9). Over 30% of the OP visits happened in secondary/primary level hospitals in Shanghai and Beijing, followed by Fuzhou (21.4%), and Chongqing relied heavily on tertiary hospitals (97.4%). Chongqing had the highest average total expense per outpatient visit ($80) with a 65% reimbursement rate. The average expense per outpatient visit was around $50 in Shanghai and Fuzhou. However, Shanghai had the lowest OOP payments and average annual expense for outpatient visits ($170) (Table 4).

### Use of health services for different age groups

Younger patient group had more hospital admissions but shorter hospitalization stays and OP visits per year. Tertiary hospitals were more preferred by the young for hospitalization but the elderly relied more heavily on tertiary hospitals for OP visits. Except for Chongqing, the elderly had higher average cost per admission. In the meantime, the elderly had lower average total expense per outpatient visit in Chongqing and Fuzhou. However, the annual total expense, including hospital admission and OP visit, was lower in the elderly in the 4 cities (Table 5).

**Table 5:** Utilization and expense of hospital admission and OP visit in 2008 for different age groups

Service type	Use of inpatient services								Use of outpatient services							
City	Shanghai		Beijing		Fuzhou		Chongqing		Shanghai		Beijing		Fuzhou		Chongqing	
Age group	<60	≥60	<60	≥60	<60	≥60	<60	≥60	<60	≥60	<60	≥60	<60	≥60	<60	≥60
# patients	240	332	223	338	85	270	175	433	215	288	119	260	60	168	103	238
# of visits	808	954	418	640	345	806	589	1158	5619	5904	3145	6987	1055	3595	1427	3669
Average number of visits per year	3.4	2.9	1.9	1.9	4.2	2.9	3.4	2.7	26.1	20.5	26.1	27.0	16.2	21.9	13.9	15.4
Per admission																
Level of hospital (%)																
Tertiary	67.1	60.0	85.2	79.2	99.1	95.3	96.8	94.6	64.1	67.5	73.3	63.4	93.6	74.3	96.8	97.7
Secondary/Primary	32.9	40.0	14.8	20.8	0.9	4.7	3.2	5.4	35.9	32.5	26.7	36.6	6.4	25.7	3.2	2.3
Average length of stay (days)	12.0	15.1	22.7	24.8	19.7	22.0	18.6	28.1	/	/	/	/	/	/	/	/
Average total expense($)	1478	1718	3625	3754	2601	3012	2681	2491	54	58	/	/	49	45	97	74
Average OOP payments($)	262	249	572	482	647	715	1078	802	7	8	/	/	16	13	30	21
Average reimbursement rate (%)	81.7	84.7	88.0	89.1	74.7	74.4	59.8	65.9	89.2	90.6	/	/	64.7	67.8	60.1	66.5
Average expense per day($)	183	165	217	236	157	157	186	136	/	/	/	/	/	/	/	/
Per Year																
Average annual days of hospital admission	40.5	43.3	42.6	47.0	80.1	64.9	62.8	74.7	/	/	/	/	/	/	/	/
Average annual expense($)	4975	4937	6796	7109	10659	8958	9022	6661	1415	1193	1304	1350	862	955	1339	1140
Average annual OOP payments($)	882	716	1073	913	2693	2113	3627	2145	190	155	698	544	279	276	410	331
Average annual reimbursement rate (%)	81.4	85.6	86.5	88.5	75.6	74.5	56.2	63.6	87.4	88.8	41.4	54.1	51.5	49.6	44.2	49.4

### Social economic development, financial burden of health care and financial protection from insurance

The medical expenses were heavy burdens for insured cancer patients. Shanghai, with the lowest total medical expense among study cities, still had an average annual cost of $6086, followed by Chongqing and Beijing ($8000). Fuzhou had the highest annual medical expenses of $10,000. Fuzhou and Chongqing relied more on hospital admission thus only 7–10% total expenses were contributed by outpatient visit while this proportion was over 15% in Shanghai and Beijing. OOP payments were reduced after reimbursement. Nevertheless Fuzhou and Beijing had the highest total annual expense, Chongqing led the total annual OOP payments of over $2771, followed by $2430 of Fuzhou. Insured patients in Beijing and Shanghai had the relatively lower OOP payments of $1377 and $935 respectively. The insurance reimbursement rate was highest for Shanghai and Beijing (85.0% and 82.3%, respectively), followed by Fuzhou (74.6%) and Chongqing (62.3%) (Table 6).

**Table 6:** Total economic burden and financial protection in 2008

	Shanghai		Beijing		Fuzhou		Chongqing	
	*N* = 572		*N* = 561		*N* = 355		*N* = 608	
Annual direct medical expense								
Average total direct medical expenditure($)	6086		7886		9963		8013	
Average percentage of total hospital admission expenditure (%)	80.1		84.6		93.0		89.5	
Total cost paid by insurance($)	5151		6510		7533		5243	
Average total OOP payments($)	935		1377		2430		2771	
Reimbursement rate (%)	85.0		82.3		74.6		62.3	
Socio-economic profile in 2008								
GDP per capita ($)	10,530 [30]		9257 [31]		4841 [32]		3356 [33]	
Household non-subsistence expenditure($)	4781		4080		3679		3174	
Economic burden and the financial protection of medical insurance								
Average proportion of OOP as to household non-subsistence expenditure (%)	19.6		33.7		66.0		87.3	
Proportion of households experiencing catastrophic health expenditure (%)	8.7		30.0		65.1		67.6	
Age group	<60	≥60	<60	≥60	<60	≥60	<60	≥60
Average salary/pension for employees/retirees	6061	2659	8111	/	3963	1820	3886	1702
Average total direct medical expenditure($)	6243	5973	7491	8147	11,267	9552	9810	7292
Average percentage of total hospital admission expenditure (%)	77.6	81.9	88.4	82.1	93.8	92.7	88.7	89.9
Total cost paid by insurance($)	5191	5122	6046	6816	8378	7267	5942	4963
Average total OOP payments($)	1051	850	1445	1332	2890	2285	3868	2329
Reimbursement rate (%)	83.1	86.3	81.5	82.9	74.6	74.6	57.8	64.2

The GDP per capita and the average non-subsistence expenditure, as key indicator of regional economic development and living standard, showed the same trend as the reimbursement rate and varied dramatically among the 4 cities. The proportion of households experiencing catastrophic health expenditure was 8.7% and 30% in Shanghai and Beijing, while over 65% of the insured patients with cancer experienced catastrophic health expenditure in Fuzhou and Chongqing.

Although insured patients over 60 years old had higher reimbursement rate in each city, they still had higher average OOP payment due to their higher total expenditure. Moreover, in each city, the average pension for retirees were less than half of that for employees.

## Discussion

With the development of medical technology, different types of effective treatments such as surgery and chemotherapy were available to cancer patients in China and almost all of the treatments require successive service utilization in a period of time. On average, cancer patients had at least 2 hospital admissions and 15 times of outpatient visits in the study cities of China which led to heavy economic burden of cancer patients and their family.

When it comes to the economic burden among different cities, an inconsistency between economic development and medical expenditure was observed. More specifically, Shanghai, despite the highest GDP per capita, had the lowest total medical expense ($6086) followed by Beijing ($7886) while Fuzhou ($9963) and Chongqing ($8013) had relatively high expense. Many factors may have contributed to such phenomenon.

First of all, the various insurance policies of UEBMI influenced the health seeking behavior of patients. Shanghai and Beijing provided better coverage for OP visit. As observed in our study, insured patients in Shanghai and Beijing indeed used more outpatient services than those in Fuzhou and Chongqing and might prevent some hospital admissions which further led to a lower annual total medical expense.

Secondly, the provider payment mechanism, Global Budget, has been implemented for many years in Shanghai, encouraging hospitals to improve their efficiency thus leading to shortened average length of stay [13, 14]. Meanwhile, each inpatient episode was paid by a contracted quota in Fuzhou and Chongqing, which faced the hazard that health providers might break one hospitalization into more hospital admissions and finally led to high total expense [15, 16].

Thirdly, large cities have a more developed health delivery system, which made it possible in Shanghai and Beijing to replace some of the inpatient services with outpatient services. Moreover, the primary and secondary hospitals also had better services capacity than that in Fuzhou or Chongqing. Herein, insured patients in Shanghai and Beijing might use more secondary and primary hospitals, which usually had a lower total expense.

One of the goals of universal health coverage (UHC) is to avoid substantial direct payments and catastrophic health expenditure [17, 18] and a consistency between economic development and financial protection provided by the UEBMI was observed. Shanghai and Beijing, with doubled GDP than that of Chongqing and Fuzhou, provided over 80% reimbursement rate on total medical expenses and less than 10% of insured patients experienced catastrophic health expenditure, even though Beijing had the similar total medical expense as that of Chongqing. Unfortunately in the same year, over 65% of households with cancer patient were estimated to experience catastrophic health expenditure in Chongqing and Fuzhou. In sum, financial protection provided by the insurance are greatly influenced by the social economic development via its impact on the capacity of collecting financial contribution for insurance and management capacity.

In terms of the financial protection between different age groups, although retirees enjoyed higher reimbursement rate documented by national policy, the average pension for retirees was only half of employee’s salary in each city, and insured cancer patients over 60 years were faced with relatively heavier financial burden than that of employees. We also observed that the elderly had fewer hospital admissions and used tertiary hospitals less while the average length of stay was longer. Hospital admission is less price-elastic than outpatient visit as it depends largely on the severity of the diseases. However, economic factor also plays its role. It is possible that the high expenditure for hospital admission became the barrier for the elderly. Only those with severe situation were admitted into the hospital.

Overall, UEBMI provided considerably financial protection on patients with cancer, especially in Shanghai and Beijing. Unfortunately, the high financial contribution rate and generous benefit package of UEBMI in Shanghai and Beijing was unlikely to be reciprocated by other cities. However, the cost-saving service utilization pattern such as more frequent use of outpatient visits and secondary and primary sectors in Shanghai and Beijing are inspiring lessons for the future development of insurance policy. Began with limited financial resources, hospital admission became the priority of insurance schemes and were entitled with high reimbursement rate and potentially induced the use of hospital admission. It is right time to revisit the health need and develop insurance benefit package to promote efficient service utilization. With the aging population and increasing prevalence of non-communicable diseases, developing special reimbursement program for outpatient services of specific diseases should be scaled-up at a national level. For diseases with multiple hospital admissions, such as cancer, Chongqing provided a good example to improve the financial protection on patients that in 2010, cancer patients only needed to pay one deductible per year (before the announcement, patients had to pay deductible for each hospital admission) [19].

We also recommend the Social Health Insurance schemes to use the OOP payment instead of reimbursement rate as the key indicator when developing the insurance policy. Strengthening on the services delivery system, especially the primary and secondary hospitals, is also important to reduce the overall financial burden of patients.

The incidence and prevalence of NCDs have a predictably rising trend which urges a sustainable and equitable financing mechanism [20]. At least half of UEBMI patients with cancers in the study were retirees. Considering their comparatively limited capacity to pay and exacerbated health needs, retirees, especially retirees with NCDs, should enjoy lower deductible and copayment rate than that of employees.

Comparing to UEBMI, URBMI and NCMS have lower capacity in collecting financial contribution. When choosing the path to increase its financial protection level, special attentions need to be paid to the efficiency of URBMI and NCMS. The insurance programme for catastrophic diseases (IPCD) was recently introduced as an extension of URBMI and NCMS and commercial insurance companies were encouraged to get involved into the operation of IPCD. It can be regarded an important leverage and opportunity to narrow the disparities between UEBMI to URBMI and NCMS and attentions should be paid to its influence on both access and affordability of services.

Due to lack of data of high quality from the other two social health insurance, our study only performed analysis on UEBMI insured cancer patients. As UEBMI had the best benefit package among the three social health insurance, our study underestimated the financial burden of cancer patients in China. Although a randomized sampling method was employed, the stage of cancer and outcome of study population were not available, which may have potential uncertain effects on our study. We may also underestimated the OP visits since study population may not use their insurance card during the deductible period (insured patients have to pay certain amount of expense through out-of-pocket payment before they get reimbursement from insurance). However, these missing records only took small proportion of total medical expenditure that wouldn’t lead to major bias on our results.

## Conclusion

Our findings show that social economic development was not necessarily associated with level of total medical expense for cancer care, but it determined the content of financial protection and health seeking behavior through its impacts on health insurance policies and mobilization and allocation of resources. The economic burden of insured cancer patients was considerably reduced by UEBMI, particularly in Shanghai and Beijing. It is the right time for health insurance schemes in China to develop sustainable financing mechanisms that can provide better financial protections and improve affordability of healthcare for insured cancer patients in China.

## Footnotes

1 We use US dollar as the currency of this research, and the exchange rate in 2008 is: 1 USD = 6.94 CNY

2 Elderly: in this study, people aged over 60 years old were categorized as elderly. Same stratification on age groups applied to the whole manuscript.
